# Establishment of a reverse genetics system for Schmallenberg virus, a newly emerged orthobunyavirus in Europe

**DOI:** 10.1099/vir.0.049981-0

**Published:** 2013-04

**Authors:** Richard M. Elliott, Gjon Blakqori, Ingeborg C. van Knippenberg, Elina Koudriakova, Ping Li, Angela McLees, Xiaohong Shi, Agnieszka M. Szemiel

**Affiliations:** Biomedical Sciences Research Complex, School of Biology, University of St Andrews, North Haugh, St Andrews, KY16 9ST, Scotland, UK

## Abstract

Schmallenberg virus (SBV) is a newly emerged orthobunyavirus that has caused widespread disease in cattle, sheep and goats in Europe. Like other orthobunyaviruses, SBV is characterized by a tripartite negative-sense RNA genome that encodes four structural and two non-structural proteins. This study showed that SBV has a wide *in vitro* host range, and that BHK-21 cells are a convenient host for both SBV propagation and assay by plaque titration. The SBV genome segments were cloned as cDNA and a three-plasmid rescue system was established to recover infectious virus. Recombinant virus behaved similarly in cell culture to authentic virus. The ORF for the non-structural NSs protein, encoded on the smallest genome segment, was disrupted by introduction of translation stop codons in the appropriate cDNA, and when this plasmid was used in reverse genetics, a recombinant virus that lacked NSs expression was recovered. This virus had reduced capacity to shut-off host-cell protein synthesis compared with the wild-type virus. In addition, the NSs-deleted virus induced interferon (IFN) in cells, indicating that, like other orthobunyaviruses, NSs functions as an IFN antagonist, most probably by globally inhibiting host-cell metabolism. The development of a robust reverse genetics system for SBV will facilitate investigation of its pathogenic mechanisms as well as the creation of attenuated strains that could be candidate vaccines.

## Introduction

The family *Bunyaviridae* has provided many examples of emerging and re-emerging diseases, such as those caused by Sin Nombre hantavirus in North America, Rift Valley fever phlebovirus in Africa and the Arabian peninsula, Crimean–Congo hemorrhagic fever nairovirus in Turkey and severe fever with thrombocytopenia phlebovirus in China (reviewed by Elliott, 1997, 2009; Feldmann, 2011). In 2011, yet another new bunyavirus appeared, this time in Europe. Farms in Germany and the Netherlands reported a disease in cattle causing transient fever, diarrhoea, loss of condition and a reduction in milk yield. Animals recovered within a few days. Towards the end of 2011, reports of abortion, stillbirths and offspring with congenital abnormalities appeared from cattle, sheep and goat herds (Garigliany *et al.*, 2012; Tarlinton *et al.*, 2012). Researchers at the Friedrich-Loeffler Institute in Germany determined the pathogen, by metagenomic analysis, to be an orthobunyavirus of the Simbu serogroup, and named it Schmallenberg virus (SBV) after the town where the first isolation was made (Hoffmann *et al.*, 2012).

The genus *Orthobunyavirus* is one of five genera that currently comprise the family *Bunyaviridae*, the others being *Hantavirus*, *Nairovirus*, *Phlebovirus* and *Tospovirus* (Plyusnin *et al.*, 2012). Bunyaviruses are characterized by the possession of a tripartite negative-sense ssRNA genome that encodes four structural proteins. The large (L) segment encodes the viral RNA-dependent RNA polymerase or L protein, the medium (M) segment encodes the virion glycoproteins Gn and Gc, and the small (S) segment encodes the nucleoprotein (N). Non-structural proteins are encoded by some viruses on the M (NSm protein) and by some on the S (NSs protein) segment. There are differences in the sizes of RNAs and proteins in viruses belonging to the different genera, and in addition all viruses within a genus share the same consensus terminal sequence on each RNA segment of the genome (Plyusnin & Elliott, 2011; Plyusnin *et al.*, 2012). The genus *Orthobunyavirus* contains at least 170 named viruses that have been conveniently divided among 18 serogroups on the basis of cross-reactivity in complement fixation, neutralization and haemagglutination-inhibition assays (Bishop, 1990; Calisher, 1996; Elliott & Blakqori, 2011). The Simbu serogroup contains 25 viruses, of which Akabane, Aino, Sathuperi, Peaton, Shamonda and Shuni viruses are known to be teratogenic in ruminants in Africa, Asia and Australia (Calisher, 1996). SBV represents the first Simbu serogroup virus that has been reported in Europe. Simbu serogroup viruses are transmitted by midges and mosquitoes, and analysis of midges trapped in Denmark and Belgium has shown evidence of SBV infection (De Regge *et al.*, 2012; Rasmussen *et al.*, 2012). SBV appears to have survived the 2011/2012 winter in Europe, and at least 12 European countries have now reported SBV infections, involving around 5000 holdings (Beer *et al.*, 2013; European Food Safety Authority, 2012). No vaccine is yet available.

Previous work from our laboratory established a reverse genetic system for the prototype orthobunyavirus, Bunyamwera virus (BUNV) (Bridgen & Elliott, 1996; Lowen *et al.*, 2004), using the bacteriophage T7 RNA polymerase system, whereby infectious virus could be generated entirely from cDNA clones. Subsequently, the system has been applied to the orthobunyavirus La Crosse virus (LACV) and the phlebovirus Rift Valley fever virus (RVFV) (Billecocq *et al.*, 2008; Blakqori & Weber, 2005; Gerrard *et al.*, 2007; Habjan *et al.*, 2008; Ikegami *et al.*, 2006). In addition, recovery of Akabane virus has been achieved using the RNA polymerase I approach (Ogawa *et al.*, 2007). One exploitation of reverse genetics is the creation of genetically engineered recombinant viruses that have potential as candidate vaccines (Bird *et al.*, 2011; Brennan *et al.*, 2011; Lowen *et al.*, 2005). Therefore, as a future tool to develop vaccine strains for SBV, we have reported here the establishment of an efficient reverse genetic system.

## Results and Discussion

### Growth of SBV in mammalian cell lines

The initial stock of SBV (isolate BH80/11-4) had been passaged once in *Culicoides* KC cells and twice in baby hamster kidney (BHK) cells. An aliquot was used for plaque purification in BHK-21 cells and working stocks were grown in BHK-21 cells for further study. The *in vitro* host range was investigated in a range of cell lines infected at both high (5 p.f.u. per cell) and low (0.05 p.f.u. per cell) m.o.i, by incubating the infected cells at 37 °C for 48 h and then titrating the yield of virus on BHK-21 cells (Table 1). SBV grew productively in all cell lines tested, irrespective of the species of origin, although with varying degrees of efficiency. The highest titres of virus were obtained in BHK-21 (hamster) and HuH7 (human) cells after infection at low m.o.i.; slightly lower titres were achieved in these cells when infected at high m.o.i. Vero E6 (monkey) and CPT-Tert (sheep) cells also generated high yields of virus, although in Vero E6 cells no difference in yield was observed between high and low m.o.i., whereas in the sheep cells the yield was greater from cells infected at high m.o.i. The other human cell lines were less productive and, interestingly, the Madin–Darby bovine kidney (MDBK) (bovine) cell line was also less efficient for SBV growth.

**Table 1.** Growth of SBV in various mammalian cell lines

Cell line (host)	Yield from high m.o.i.*	Yield from low m.o.i.**†**	Plaque phenotype**‡**
BHK-21 (hamster)	3.0×10^7^	1.1×10^8^	3 mm, clear
BSR-T7/5 (hamster)	8.5×10^5^	1.5×10^7^	1 mm, clear
A549 (human)	1.4×10^6^	3.0×10^5^	1 mm, clear
HuH7 (human)	1.1×10^7^	1.0×10^8^	1–2 mm, cloudy
HeLa (human)	6.0×10^5^	4.5×10^4^	Indistinct
Hep2 (human)	7.0×10^3^	6.5×10^3^	Indistinct
MDBK (cow)	4.5×10^5^	9.0×10^3^	Indistinct
MDCK (dog)	1.4×10^5^	6.0×10^6^	0.5 mm, clear
Vero E6 (monkey)	1.1×10^7^	1.1×10^7^	1 mm, clear
CPT-Tert (sheep)	3.0×10^7^	3.0×10^6^	1.5–2 mm, clear

* Yield in p.f.u. ml^−1^ from cells infected at an m.o.i. of 5 and titrated in BHK-21 cells.

† Yield in p.f.u. ml^−1^ from cells infected at an m.o.i. of 0.05 and titrated in BHK-21 cells.

‡ Size and appearance of plaques after 3 days incubation at 37 °C.

Previous studies on BUNV and Marituba orthobunyavirus (Kascsak & Lyons, 1978; Volkmer *et al.*, 1983) showed yields of virus from mammalian cells to be multiplicity dependent, and that the ease with which defective-interfering particles were generated was cell dependent. Thus, for BUNV, virus yields were greater from BHK-21 and Vero cells infected at low m.o.i. than at high m.o.i., whereas for MDBK cells, yields were directly correlated with the input multiplicity. SBV behaved similarly to BUNV in BHK-21 and MDBK cells, although not in Vero E6 cells (Table 1); the generation of defective-interfering particles was not investigated.

**Fig. 1.**
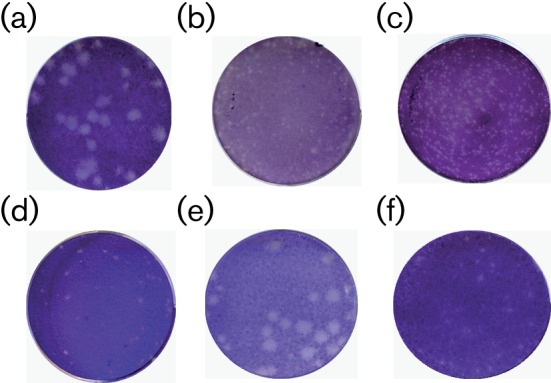
Plaque phenotypes of SBV in different cell lines. Infected cells were fixed after 3 days incubation at 37 °C and stained with crystal violet. (a–d) Wild-type (authentic) SBV in BHK-21 (a), BSR-T7/5 (b), Vero E6 (c) and CPT-Tert (d) cells; (e) rSBV in BHK-21 cells; (f) rSBVdelNSs in BHK-21 cells.

The ability of SBV to form plaques in the different cell lines was also studied. Marked differences in plaque morphology were noted, with the largest and clearest plaques produced in BHK-21 cells (Fig. 1a–d). Plaques were much smaller in Vero E6 cells, a line that is used routinely to propagate orthobunyaviruses. Of note was the fact that plaques were also smaller in BSR-T7/5 cells, a BHK cell-derived line that constitutively expresses T7 RNA polymerase (Buchholz *et al.*, 1999). Easily discernible plaques were also formed in CPT-Tert cells, although in all other tested cell lines plaques were very small after 3 days (Table 1). Therefore, for routine use, plaque assays were carried out in BHK-21 cells.

### Cloning the SBV genome

**Fig. 2.**
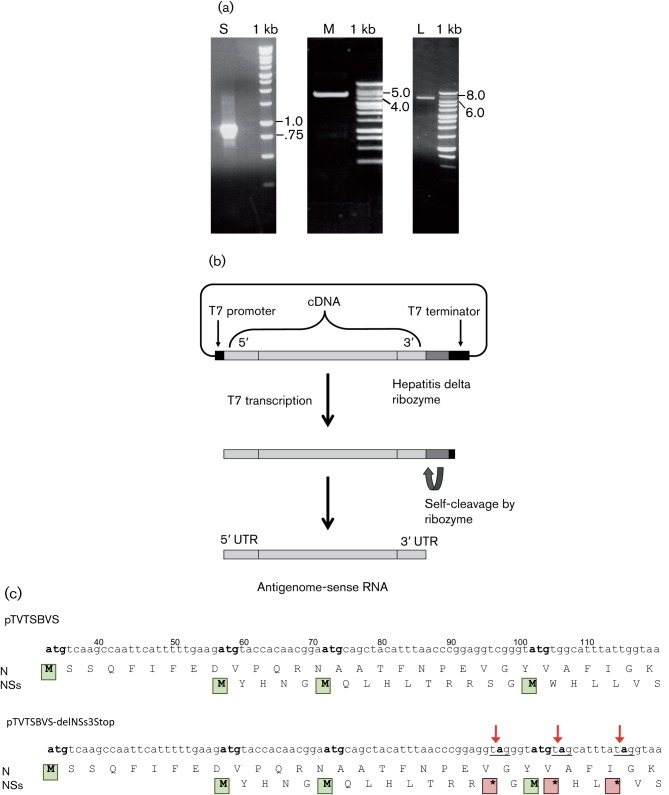
Production of cDNA clones. (a) Full-length RT-PCR products derived from the S, M and L segments as indicated. The amplified DNAs were separated by electrophoresis on agarose gels along with DNA markers (1 kb ladder; Promega), with the relevant fragment sizes indicated. (b) Schematic of the pTVT series of plasmids (see Methods), showing transcription by T7 RNA polymerase and processing by the hepatitis delta ribozyme to generate antigenome-sense SBV RNAs having the correct 3′ ends. (c) S segment cDNA sequences. The nucleotide sequence encompassing the N and NSs start codons (methionine, M) is shown and the positions of the mutations used to introduce translational stop codons (asterisks) in the NSs ORF are indicated. Numbers are nucleotide positions in the full-length S segment cDNA.

The sequences of the SBV genome segments (isolate BH80/11-4) (GenBank accession nos HE649912–HE649914; Hoffmann *et al.*, 2012) mostly lack the terminal consensus nucleotides characteristic of orthobunyavirus RNAs (Plyusnin *et al.*, 2012). For the M segment, the 3′ consensus sequence is present in the database entry but there are additional nucleotides thereafter, which we presumed to be artefacts generated during the assembly process to produce full-length sequences. By comparison with the available sequences in the databases for other Simbu serogroup viruses, the missing bases were predicted, and segment-specific oligonucleotides were designed (Table S1, available in JGV Online) to amplify complete cDNAs by RT-PCR. Amplified DNA products of approximately 0.8, 4.4 and 6.9 kb were obtained (Fig. 2a) and cloned into plasmid TVT7R(0,0) (Johnson *et al.*, 2000) such that the antigenome-sense RNA would be transcribed by T7 RNA polymerase. The complete nucleotide sequences of the cDNA clones were determined and exactly matched the database sequences.

The plasmids were then used in a three-plasmid rescue system (Lowen *et al.*, 2004) by transfection into T7 RNA polymerase-expressing BSR-T7/5 cells. Full-length antigenome-sense RNAs having exact ends are transcribed in the cytoplasm of these cells and, although apparently neither 5′ capped nor 3′ polyadenylated, the RNAs can be translated to give viral proteins. Presumably, the N protein is then able to interact with the RNAs to generate ribonucleoproteins, which can be replicated by the expressed SBV polymerase to initiate the infectious cycle. The supernatant medium was harvested at 5 days post-transfection and used in a plaque assay in BHK-21 cells. Cells transfected with the mixture of three plasmids yielded a titre of 1.15×10^6^ p.f.u., whereas no virus was recovered from a transfection lacking the L segment cDNA. The plaque morphology of the recombinant virus (rSBV) was indistinguishable from that of authentic virus (Fig. 1e). The rescue of SBV from cDNA was repeated several times and always generated virus. The complete nucleotide sequence of rSBV was determined and matched that of the parental plasmids. Thus, SBV, like BUNV, LACV and RVFV, can be recovered from cDNAs expressing full-length antigenome-sense RNAs without the need for ‘helper’ plasmids that express viral proteins independently (Elliott, 2012).

The NSs proteins of BUNV and LACV are the major virulence determinants and act as interferon (IFN) antagonists by blocking cellular RNA polymerase II-driven transcription (reviewed by Elliott & Blakqori, 2011). To determine whether the NSs protein of SBV might have a similar role, a recombinant virus was created lacking NSs expression. Previously, we generated an NSs deletion mutant of BUNV by mutation of the NSs start codon, but this resulted in either expression of NSs-related proteins initiated from AUG codons downstream of the authentic start codon or the generation of a mutation that introduced a new AUG codon, resulting in an N-terminally deleted NSs protein (Hart *et al.*, 2009; van Knippenberg *et al.*, 2010). Therefore, a different strategy was used whereby three translational stop codons were introduced in the NSs ORF at codons 14, 17 and 20 (C→A at nt 96, G→A at nt 10, and T→A at nt 115 in the S segment) such that the amino acid sequence of the overlapping N protein remained unchanged (Fig. 2b). This scheme would allow translation initiation at the NSs start codon and potentially the production of an 11 aa peptide only, but there should not be further ribosome scanning to downstream AUG codons. The NSs-deleted S segment cDNA was co-transfected with L- and M-segment cDNAs into BSR-T7/5 cells, and the medium (containing recombinant virus, which was named rSBVdelNSs) was harvested 5 days after transfection for use in a plaque assay in BHK-21 cells. The plaques were stained after 3 days and were noticeably smaller and less distinct than plaques produced by the wild-type virus (Fig. 1f). The yield was about 1×10^5^ p.f.u. The nucleotide sequence of the recovered virus was determined and the mutations in the S segment were confirmed (Fig. 2c).

### Growth properties of authentic and recombinant viruses in mammalian cells

**Fig. 3.**
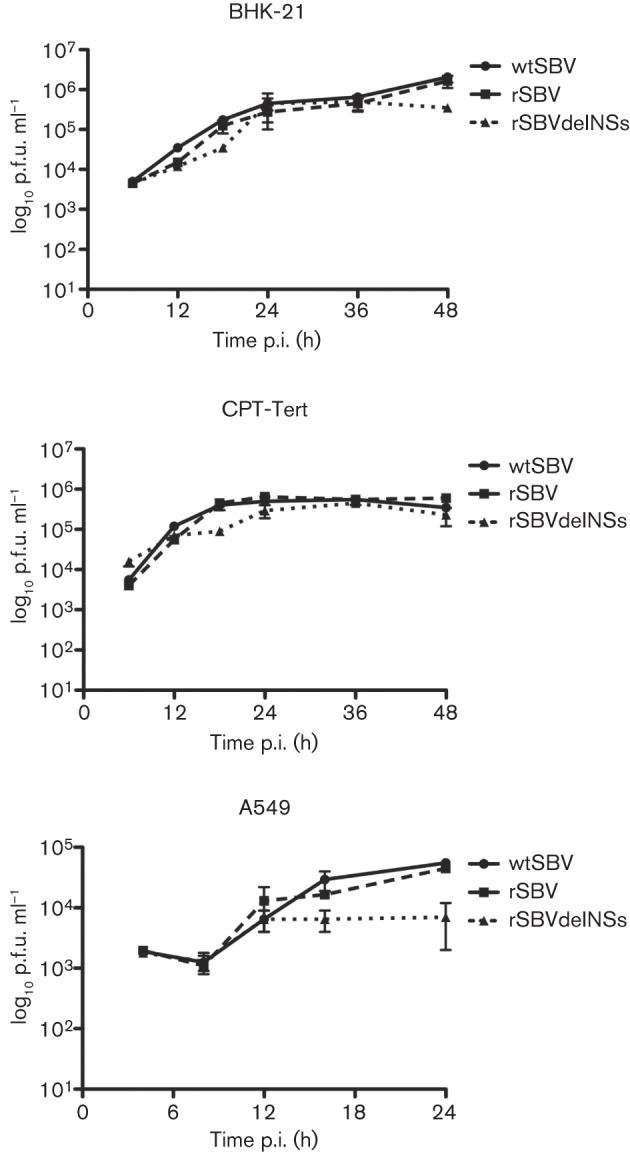
Virus growth curves. BHK-21, CPT-Tert or A549 cells were infected with wtSBV, rSBV or rSBVdelNSs at an m.o.i. of 3 and incubated at 37 °C. The titre of virus released into the supernatant at different times post-infection (p.i.) was determined by plaque assay in BHK-21 cells.

Growth curves of authentic SBV (hereafter designated wtSBV), rSBV and rSBVdelNSs were compared in BHK-21, CPT-Tert and A549 cells infected at an m.o.i. of 3 and maintained at 37 °C (Fig. 3). Essentially no difference in growth kinetics was observed between wtSBV and rSBV in any of the cell lines, although the growth in A549 cells was less efficient than in the other two lines. In BHK-21 cells, rSBVdelNSs grew similarly to wtSBV or rSBV until 36 h p.i. but did not achieve as high a titre at 48 h p.i., whereas in CPT-Tert cells the mutant virus grew slightly slower than wtSBV or rSBV. However, in A549 cells, which are known to have a competent IFN system, the growth of rSBVdelNSs appeared to be more restricted after 12 h p.i., and the titre at 24 h p.i. was more than tenfold lower than the genetically intact viruses.

### Protein synthesis by authentic and recombinant viruses in mammalian cells

**Fig. 4.**
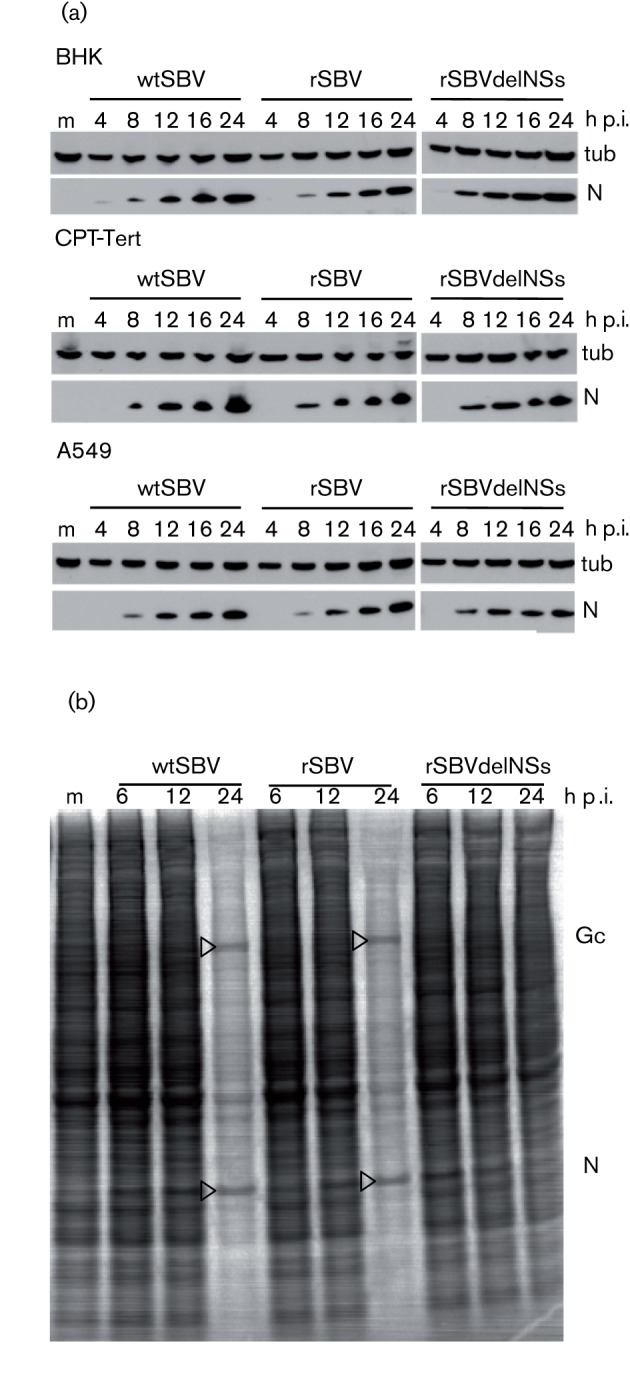
Viral protein synthesis in mammalian cells. (a) N production. BHK-21, CPT-Tert or A549 cells were infected with wtSBV, rSBV or rSBVdelNSs at an m.o.i. of 3 and incubated at 37 °C. Cell lysates were prepared at different times after infection, and proteins were separated by SDS-PAGE and transferred to nitrocellulose membrane. Accumulation of viral N was detected by reaction with anti-N antibody, and detection of tubulin (tub) was used as a loading control. (b) Host-cell protein shut-off. BHK-21 cells were infected with wtSBV, rSBV or rSBVdelNSs at an m.o.i. of 3 and incubated at 37 °C. At different times p.i., the cells were labelled for 1 h with [^35^S]methionine and cell extracts were fractionated by SDS-PAGE. The dried gel was exposed to X-ray film. The positions of viral Gc and N are indicated. m, Mock-infected cells.

The production of viral N protein by the different viruses in BHK-21, CPT-Tert and A549 cells was monitored by Western blotting using a monospecific antibody raised against bacterially expressed SBV N protein; detection of tubulin was used as a loading control (Fig. 4a). N was clearly detected at 8 h p.i. in all cell lines infected with all viruses. Although the blots were not quantitative, the amount of N appeared to be slightly lower in A549 cells compared with BHK-21 or CPT-Tert cells. Overall, no marked differences in the kinetics of N accumulation were seen in cells infected with wtSBV or the recombinant viruses.

BHK-21 cells infected with the three viruses were also radiolabelled at different times after infection with [^35^S]methionine and cell extracts were analysed by SDS-PAGE. Both authentic and recombinant wild-type viruses induced significant shut-off of host-cell protein synthesis by 24 h p.i., and two prominent bands most likely corresponding to the viral Gc and N proteins were apparent. No shut-off was observed in cells infected with rSBVdelNSs (Fig. 4b). Thus, the accumulated data indicated that the mutant virus lacking NSs behaved similarly to other orthobunyaviruses that do not produce the S-segment-encoded NS protein.

### Replication in mosquito cells

**Fig. 5.**
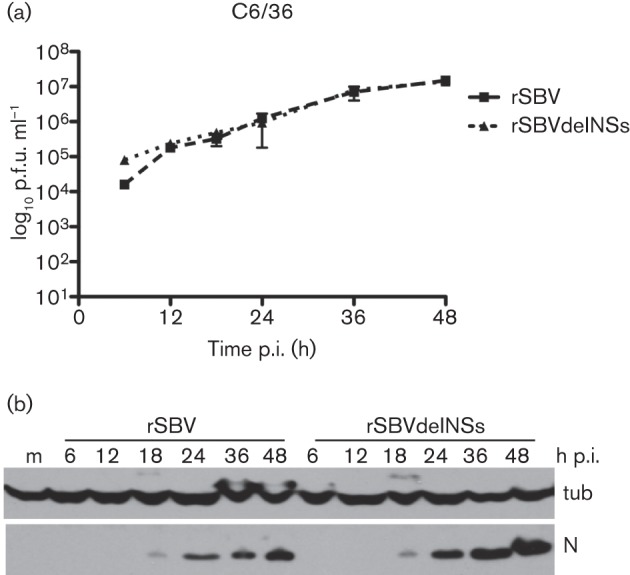
Replication in mosquito cells. (a) Growth kinetics. *A. albopictus* C6/36 cells were infected with rSBV or rSBVdelNSs at an m.o.i. of 3 and incubated at 28 °C. The amount of virus released into the supernatant at different times p.i. was determined by plaque assay in BHK-21 cells. (b) N production. Cells were infected as above and lysates were prepared at different times p.i. Proteins were separated by SDS-PAGE and transferred to nitrocellulose membrane. Accumulation of viral N protein was detected by reaction with anti-N antibody, and detection of tubulin (tub) was used as a loading control. m, Mock-infected cells.

Other Simbu group viruses have been isolated from both culicoid midges and various species of mosquito (Calisher, 1996). SBV has been shown to grow in *Culicoides variipennis* larvae cells (KC cells; Hoffmann *et al.*, 2012), and here we investigated whether cultured mosquito cells would also be permissive. Therefore, we infected *Aedes albopictus* C6/36 cells with rSBV or rSBVdelNSs, titrated the virus released into the supernatant by plaque assay and monitored the production of viral N by Western blotting. Both viruses grew with similar kinetics, achieving titres in excess of 10^7^ p.f.u. ml^−1^ by 48 h p.i. (Fig. 5a). However, no cytopathic effect (CPE) was apparent in the cells at any stage after infection with either virus. N was first detected at 18 h p.i. for both viruses, and accumulated thereafter in a similar manner (Fig. 5b). These results indicated that SBV can grow productively in *A. albopictus* C6/36 cells and that the NSs protein is not required, and thus SBV behaves similarly to BUNV in this cell line (Szemiel *et al.*, 2012). The ability of SBV to replicate in other mosquito cell lines or to replicate in and be transmitted by live mosquitoes awaits further investigation.

### IFN induction by recombinant viruses

**Fig. 6.**
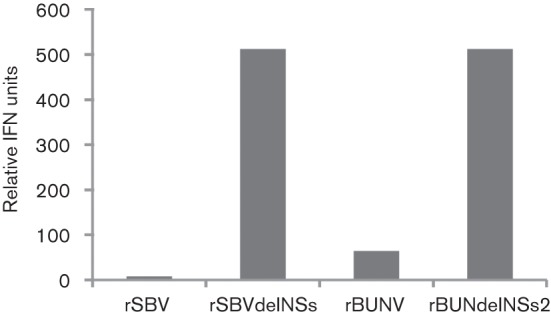
IFN bioassay. A549 cells were infected with wtSBV, rSBVdelNSs, wtBUNV or rBUNdelNSs at an m.o.i. of 1 and incubated at 37 °C for 48 h. Twofold dilutions of the clarified and UV-treated supernatant were used to treat fresh A549 cells in a 96-well plate for 24 h. The cells were then infected with EMCV and the development of CPE was monitored at 48 h p.i. by staining with crystal violet. The production of IFN was calculated according to the highest dilution of supernatant giving protection against EMCV infection and is expressed as relative IFN units. The experiment was conducted twice and gave identical results.

A biological assay used previously to monitor IFN production in response to orthobunyavirus infection (Mohamed *et al.*, 2009) was employed here to investigate IFN induction by rSBV and rSBVdelNSs. A549 and CPT-Tert cells were infected with recombinant viruses and UV-inactivated medium from the cells was used to treat fresh A549 or CPT-Tert reporter cells; if IFN was present in the medium, an antiviral state would be induced and the cells would be protected from subsequent infection with encephalomyocarditis virus (EMCV). Recombinant BUNV (rBUNV) and BUNV with the NSs deleted (rBUNVdelNSs2 ) were used as controls. The relative amounts of IFN produced were calculated according to the highest dilution of supernatant affording protection to the cells from EMCV infection. As shown in Fig. 6, medium from A549 cells infected with wild-type viruses showed relatively little IFN production. By contrast, medium from rSBVdelNSs- or rBUNVdelNSs2-infected cells largely protected the reporter from EMCV infection, indicating significant induction of IFN in the initial infections. Thus, rSBVdelNSs, like its BUNV counterpart rBUNVdelNSs2 lacking NSs, is an IFN inducer, implying that the NSs protein functions as an IFN antagonist. However, no protection against EMCV infection was noted in sheep cells treated with medium from either rSBVdelNSs- or rBUNVdelNSs2-infected CPT-Tert cells (data not shown), indicating that CPT-Tert cells are incapable of producing IFN, although they have previously been shown to respond to exogenously applied IFN (Ratinier *et al.*, 2011).

### Concluding remarks

SBV has a typical orthobunyavirus genetic organization and behaves similarly to many other orthobunyaviruses in terms of growth characteristics in mammalian cell lines. Wild-type SBV caused shut-off of host-cell protein synthesis in mammalian cells and, as described previously for BUNV and LACV, this property was associated with expression of the NSs protein. In addition, in IFN-competent cells, the NSs-deleted virus induced IFN production, whereas this was not the case in wild-type virus-infected cells. Thus, NSs acts as the viral IFN antagonist.

SBV is still spreading throughout Europe, although the full extent will not be apparent until the 2012/2013 lambing season. Experience with other Simbu serogroup viruses suggests that vaccination could be a useful control measure (Kim *et al.*, 2011). The relative ease with which a reverse genetics system was established for SBV, and the robustness of both wild-type and NSs-deleted virus recovery, indicate that the system can be exploited to generate further recombinant viruses that could be potential live-attenuated vaccines. Previously, we described how rearrangement of the non-coding/coding sequences (Lowen *et al.*, 2005) or deletions within the non-coding sequences (Lowen & Elliott, 2005; Mazel-Sanchez & Elliott, 2012) of the BUNV genome attenuated virus replication. Similar approaches are under investigation in order to generate attenuated SBV strains.

## Methods

### 

#### Cells.

BHK-21 cells were grown in Glasgow’s minimal essential medium (GMEM) supplemented with 10 % tryptose phosphate broth (TPB) and 10 % newborn calf serum (NCS). BSR-T7/5 cells, which stably express T7 RNA polymerase (Buchholz *et al.*, 1999), were provided by K.-K. Conzelmann (Max-von-Pettenkofer Institut, Munich, Germany) and grown in GMEM supplemented with 10 % TPB, 10 % FCS and 1 mg G418 ml^−1^. CPT-Tert cells (Arnaud *et al.*, 2010) are sheep choroid plexus cells immortalized with the simian virus 40 T-antigen and human telomerase reverse transcriptase and were kindly provided by Dr David Griffiths (Moredun Research Institute, Edinburgh, UK); they were grown in Iscove’s modified Dulbecco’s medium supplemented with 10 % FCS. A549, Vero E6 and HeLa cells were maintained in Dulbecco’s modified Eagle’s medium (DMEM) supplemented with 10 % FCS, and MDBK, MDCK, Hep2 and HuH7 cells were grown in DMEM containing 10 % FCS and 1 % non-essential amino acids. All mammalian cell lines were grown at 37 °C with 5 % CO_2_. *A. albopictus* C6/36 cells (Igarashi, 1978) were maintained at 28 °C in Leibovitz 15 medium supplemented with 10 %FCS and 10 % TPB.

#### Viruses.

The BH80/11-4 isolate of SBV was generously provided by Dr Martin Beer (Friedrich-Loeffler Institute, Insel Reims, Germany) and was propagated and plaque purified in BHK-21 cells using standard procedures (Mazel-Sanchez & Elliott, 2012), as were recombinant SBVs generated from cDNA clones. rBUNV and the NSs-deleted version, rBUNVdelNSs2 (Hart *et al.*, 2009), were grown and titrated in BHK-21 cells. Viruses were titrated by plaque assay in the different cell lines under an overlay comprising GMEM supplemented with 2 % NCS and 0.6 % Avicell (FMC) and incubated at 37 °C for 3 days. Cell monolayers were fixed with 4 % formaldehyde and plaques were visualized by staining with crystal violet.

#### Cloning of SBV cDNA.

SBV was grown in BHK-21 cells at 37 °C until a marked CPE was noted, and 1.75 ml clarified supernatant medium was concentrated approximately tenfold using an Amicon Ultra-15 centrifugal filter device (Millipore). RNA in the concentrated sample was extracted using a QIAamp Viral RNA Mini kit according to the manufacturer’s instructions, and the RNA was eluted in 60 µl AVE buffer (Qiagen). First-strand cDNA was synthesized from 5 µl RNA using 100 pmol segment-specific forward primer (Table S1) and either Moloney murine leukemia virus (for the S segment; Promega), Finzymes Phusion (for the M segment; New England Biolabs) or Transcriptor (for the L segment; Roche) reverse transcriptase according to the respective manufacturer’s instructions. Segment-specific cDNAs were amplified by PCR using appropriate primer pairs (Table S1) with optimized cycling conditions (available from the authors on request) and KOD polymerase (for the S and L segments; Merck) or Phusion polymerase (for the M segment; New England Biolabs). Full-length PCR products were purified by agarose gel electrophoresis, digested with *Bsm*BI (S and M cDNA) or *Bfu*AI (L cDNA) and ligated into *Bbs*I-linearized plasmid TVT7R(0,0) (Johnson *et al.*, 2000). The cDNA inserts included an extra G residue at their 5′ ends for efficient T7 transcription, and the inserts were cloned such that T7 polymerase would transcribe antigenome-sense RNAs. The plasmids were named pTVTSBVL, pTVTSBVM and pTVTSBVS. To abolish NSs expression from the S segment, three point mutations were introduced into pTVTSBVS (C96A, G106A and T115A) using a QuikChange Site-directed Mutagenesis kit (Stratagene) using primers SBdNSs3ST_FW and SBdNSs3ST_RV (Table S1). These substitutions created in-frame translation stop codons at positions 14, 17 and 20 in the NSs ORF (Fig. 2c), and the plasmid was designated pTVTSBVS-delNSs3Stop.

#### Generation of recombinant viruses from cDNAs.

Recombinant viruses were produced using a three-plasmid rescue system (Lowen *et al.*, 2004). Subconfluent BSR-T7/5 cells (3×10^5^ cells per well of a six-well plate) were transfected with 1 µg each of pTVTSBVL, pTVTSBVM, and pTVTSBVS or pTVTSBVS-delNSs3Stop using 5 µl Lipofectamine 2000 (Invitrogen) in a total volume of 500 µl OptiMEM (Invitrogen). Transfected cells were incubated at 37 °C and the supernatants were harvested when a marked CPE was observed (5 days post-transfection). The rescue outcome was assessed by plaque assay on BHK-21 cells. The genome segments of the recovered viruses were amplified by RT-PCR and their nucleotide sequences determined.

#### Antibodies.

The coding sequence for SBV N was amplified by PCR from pTVTSBVS with oligonucleotides SBV-N-*Sac*I-tev and SBV-N-r-*Xho*I (Table S1), and cloned into modified pDEST14 vector (Invitrogen) using *Sac*I and *Xho*I restriction sites, generating plasmid p14SBVN. The N ORF has an N-terminal hexahistidine (6His) tag and a tobacco etch virus protease site (for removal of the 6His tag). Proteins were expressed in *Escherichia coli* BL-21 Rosetta 2 cells (Merck) following induction with IPTG (1 mM final concentration) at 20 °C for 16 h with shaking. Recombinant proteins were purified by binding to Ni-NTA resin, eluted with 200 mM imidazole, 0.3 M NaCl, 0.1 M Tris/HCl (pH 8.0), and concentrated into PBS containing 5 % glycerol using a Vivaspin 20 centrifugal concentrator (Sartorius AG). Purified proteins were used to generate polyclonal rabbit antisera commercially (Eurogentec). Anti-tubulin antibody (clone B512) was purchased from Sigma. Both antibodies were used at a 1 : 5000 dilution in Western blotting experiments.

#### Viral protein synthesis.

BHK-21 cells were infected at an m.o.i. of 3 p.f.u. per cell, incubated at 37 °C and cell lysates were prepared at various times p.i. by the addition of 300 µl lysis buffer [100 mM Tris/HCl (pH 6.8), 4 % SDS, 20 % glycerol, 200 mM DTT, 0.2 % bromophenol blue and 25 U ml^−1^ Benzonase (Novagen)]. For metabolic labelling, 35 µCi per well of TRANS^35^S-Label (MP Biomedicals) in methionine-free DMEM was added and incubation was continued for 2 h before harvest. Proteins were separated by SDS-PAGE on a 4–12 % acrylamide gel (Invitrogen). Thereafter, proteins were transferred to Hybond-C membrane for Western blotting (as described by Carlton-Smith & Elliott, 2012), or, for radiolabelled proteins, the gel was fixed, dried and exposed to X-ray film.

#### Biological assay for IFN production.

This assay was carried out essentially as described by Mohamed *et al.* (2009). In brief, A549 or CPT-Tert cells in 35 mm dishes were infected with the different viruses at an m.o.i. of 1 and incubated at 37 °C for 48 h. Supernatant fluid was clarified by centrifugation, residual virus was inactivated by UV treatment, and twofold serial dilutions of the medium were applied to fresh A549 or CPT-Tert cells grown in 96-well plates for 24 h. The cells were then infected with EMCV, which is sensitive to IFN, and incubated for 4 days at 37 °C. The cells were fixed with formaldehyde and stained with crystal violet to monitor the development of CPE. Based on the dilution affording protection against EMCV infection, the relative amount of IFN produced was calculated.

## Notes

A supplementary table is available with the online version of this paper.
